# Special Issue: “New Methods in Microbial Research 2.0”: Editorial

**DOI:** 10.3390/microorganisms11030718

**Published:** 2023-03-10

**Authors:** Juan M. Gonzalez

**Affiliations:** Institute of Natural Resources and Agrobiology of Sevilla (IRNAS-CSIC), E-41012 Sevilla, Spain; jmgrau@irnase.csic.es

Today, it is definitively accepted that microorganisms play a central role in the functioning and maintenance of our planet and the organisms thriving on it. Nevertheless, we are starting to understand both how microorganisms function and the huge microbial diversity existing on Earth. As a result, we are beginning to comprehend and apply the implications of complex microbial communities and each of their specific members in both local (i.e., local ecosystems and/or organisms) and global (i.e., whole-planet change) processes. All animals and plants thriving on Earth depend on microbial activity for their growth and maintenance. Microbial speciation and persistence are topics that are yet to be well understood. Furthermore, microbial activity and the growth of microorganisms over a broad spectrum of environmental conditions are poorly understood, and therefore, our comprehension of microbial relevance, applications, and potential management issues await further advances in microbiology.

Microbiology is a truly multidisciplinary science that is directly related to other fields that would otherwise be considered distant. For instance, at present, the medical and clinical implications of microbial diversity and activity are currently being established, with a major focus on understanding the human microbiome [1]. The role of microorganisms and their enzymes are a key component of biotechnology and its related industry [2]. Agriculture, soil and ecosystem health, environment sustainability and other environment-related aspects are directly linked to the persistence and performance of microorganisms [3]. It is difficult to imagine microorganisms and how they function without considering chemistry and physics. Life ultimately becomes a complex set of chemical reactions, and microorganisms thrive while ruled by physical laws, like all living creatures on our planet [4]. Therefore, the potential applications of enzymes as sustainable biocatalysts are very broad [5]. The detection and quantitation of compounds (e.g., organics and inorganics, biomolecules, and pollutants) using chemical procedures and novel equipment (e.g., HPLC, gas chromatography, MALDI-TOF mass spectroscopy, and NMR spectroscopy) have been essential in providing new knowledge to our understanding of microorganisms, their biochemistry and physiology, and their role in industries, clinical settings, and nature. In addition, the physical ruling mechanisms of bacterial motility at low Reynolds numbers [6] and diffusion-dependent substrate accessibility [7] have become important aspects to consider in order to understand microbial activity, growth, and their consequences and applications. The complexity of the microbial world requires both an extensive knowledge and the capability to conduct both multidisciplinary collaborations and interactions. This would allow to propose avenues for the future advancement of our understanding of microorganisms and their broad range of implications on both our daily lives and our planet’s sustainability.

It has been mentioned on numerous occasions that the advancement of the microbiological sciences (considering their broadest perspectives) has been strongly dependent on the application of new or imported methods for the study of microorganisms. Mostly, this has been considered to be true and a statement proven throughout history. From the origins of microbiology—remembering Pasteur’s advancements regarding bacterial cultures and disease analyses [8]—to the new concept of the tree of life (differentiating Archaea, Bacteria and Eukarya)—as stated by Woese [9], using phylogenetic analyses made possible by introducing nucleic acid sequencing methods and the initial steps of bioinformatics—t. The application of molecular biology techniques has resulted in major advancements in our understanding of microbial cell mechanisms. The continuously improved sequencing methods and, most importantly, the new generation sequencing techniques have been decisive tools which have allowed us to begin to understand the incredibly high amount of microbial diversity present in the world, using the great genetic potential of microorganisms through the study of their genomes and the great enhancement of all -omics fields from the end of the previous century [10]. We cannot forget the enormously wide-ranging implications of applying novel bioinformatics tools to the analysis of microbial diversity and genomics [11]; in the past half century, microbiology has experienced an unimaginable growth at a vertiginous pace. Let us hope that this trend is maintained and new applications, innovative strategies and analyses, and imported and fully newly designed methods continue to be produced. Following this trend, this Special Issue collection—“New Methods in Microbial Research”—represents a major platform from which all new related methodologies and assessments of their potential can be spread.

The Special Issue “New Methods in Microbial Research 2.0” (https://www2.mdpi.com/journal/microorganisms/special_issues/MicrobialResearch_2; accessed on 9 March 2023) is a continuation of a previous Special Issue which successfully initiated this series on novel methodologies in microbiology and microbial studies. On this occasion, there are 15 contributions of relevance present in the Special Issue which aim to advance our understanding of specific topics within the microbiological sciences. Below, these contributions are briefly summarized.

A methodology to improve the production of rhamnolipids—extensively used as biosurfactants—is presented by Matatkova et al. [12]. Culture methods to search for and optimize lipase production by bacteria from polluted environments are presented by Pham et al. [13]. Wu et al. [14] characterized the probiotic potential of lactic acid bacteria from different sources and their performances in soymilk fermentation. A set of plasmids aimed to conduct C-terminal epitope switching using restriction digestion is presented by Hayashi et al. [15] in order to be used as tools for analyzing the protein function of yeasts. An additional study by Phetburom et al. [16] involved monitoring the dissemination of mobile colistin resistance genes (*mcr*) tested throughout the pork supply chain to defy antimicrobial resistance and ensure public health. Yao et al. [17] developed techniques of fast-fluctuation-enhanced structured illumination microscopy to analyze and demonstrate the dynamic structures of septin during cytokinesis in budding yeasts. A variety of procedures to evaluate the contaminant *Bacillus cereus* in Mexican chili powder are proposed by Hernandez et al. [18] for the detection and characterization of the public health risk of this pathogen. Ricketson et al. [19] compares the use of molecular techniques (PCR) with culturing techniques used to detect *Streptococcus pneumoniae* in the respiratory tracts of children; the preference of PCR to detect the nasopharyngeal carriage of that pathogen is highlighted. The differentiation of closely related bacteria is generally a bottle-neck in a variety of discriminating microbiological scenarios; thanks to Vaitiekūnaitė et al. [20], this process can now be greatly facilitated using label-free surface-enhanced Raman spectroscopy (SERS). Sereme et al. [21] presents the development of a non-invasive immunological method for the detection of enteroviruses in gorilla feces, opening up novel prospects to analyze zoonotic infectious diseases.

In addition to the above publications, which used novel methodologies in their research, a number of review papers form part of this Special Issue. Trego et al. [22] present a study on different analytical tools used to quantify and compare diversity estimates, focusing on the interpretation of amplicon sequencing data, a technique commonly used in microbial community surveys; the fast progress in microbial informatics and the recent novel methodology in this area are essential in our understanding of the dynamics of complex microbial communities. A review [23] on the use of essential oils-loaded nanoparticles aiming to reduce specific pathogens is applied to food quality and safety, leading to their incorporation into the food industry in the near future. The application of *Enterococcus* spp. and its production of the bacteriocin named enterocin—in addition to their prospects as biopreservative of foods, mainly meat-based products—were developed and are reviewed by Kasimin et al. [24]. Khan et al. [25] reviewed novel therapeutic agents for Hepatitis B and D infections, including a variety of inhibitors that affect these hepatitis viruses’ life cycles and their usefulness in the fight against them. A review on the use of microfluidic-based techniques which allow single-cell analyses in *Mycobacterium tuberculosis* diagnostics, research, and drug discovery [26] is another interesting contribution to this Special Issue.

In summary, this Special Issue—“New Methods in Microbial Research 2.0”—includes different novel techniques and reviews of great interest that significantly contribute to disseminating the current advances in microbiology by gathering together a broad spectrum of methodologies and microbiological topics. The next Special Issue in this collection, “New Methods in Microbial Research 3.0” (https://www.mdpi.com/journal/microorganisms/special_issues/F65S66596G; accessed on 9 March 2023), is open for submission so that additional methods and developments can be shared among the scientific community. Here, we aim to contribute to the spread of novel techniques useful for the advancement of microbiology and leading to major contributions in a variety of related disciplines.

## Data Availability

Not applicable.
